# Chronic HCV infection: epidemiological and clinical relevance

**DOI:** 10.1186/1471-2334-12-S2-S2

**Published:** 2012-11-12

**Authors:** S Zaltron, A Spinetti, L Biasi, C Baiguera, F Castelli

**Affiliations:** 1 University Division of Infectious and Tropical Diseases, University of Brescia and Spedali Civili General Hospital, 25123, Brescia, Italy

## Abstract

Hepatitis C virus (HCV), first recognized as a cause of transfusion-associated acute and chronic hepatitis in 1989, plays a major role as a cause of chronic liver injury, with potential for neoplastic degeneration. It is mainly transmitted by the parenteral route. However, although with lower efficiency, it may be also transmitted by sexual intercourses and by the mother-to-child route. Epidemiological evidence shows that a wave of infection occurred in the 1945-65 period (baby boomers) in western countries. After acute infection, as many as 50-85% of the patients fail to clear the virus resulting in chronic liver infection and/or disease. It is estimated that, on a global scale, about 170 million people are chronically infected with HCV, leading to about 350.000 deaths yearly. Among western countries southern Europe, and particularly Italy, is among the most affected areas. The impact on the public health systems is noteworthy, with high number of hospitalizations due to chronic liver disease, cirrhosis or hepatocellular carcinoma. While waiting for a safe and effective vaccine to be made available, new promising direct-acting antiviral (DAA) drugs offer a better therapeutic scenario than in the past even for the poor responder genotypes 1 and 4, provided that effective screening and care is offered. However, the long and aspecific prodromic period before clinical symptoms develop is a major obstacle to early detection and treatment. Effective screening strategies may target at-risk groups or age specific groups, as recently recommended by the CDC.

## Introduction

The hepatitis C virus (HCV) is a RNA virus belonging to the Flaviviridae family. It has been first recognized as a cause of transfusion-associated acute and chronic hepatitis (formerly known as non-A, non-B hepatitis) in 1989 [1]. Six different genotypes (HCV-1 to HCV-6) and several subtypes have subsequently been identified, with different geographical and virulence patterns and different response to conventional therapy. Its epidemiological relevance as a cause of chronic liver injury, with potential for neoplastic degeneration, has since been fully appreciated. At present, it is estimated that about 170 million people, roughly 3% of the world population, are chronically infected with HCV leading to about 350.000 deaths yearly, related to complications such as cirrhosis and liver cancer [2].

During the last 2 decades, an increasing body of knowledge has accumulated as to its virological properties, modes of transmission, epidemiological characteristics, pathogenesis, clinical features and public health impact. While waiting for a safe and effective vaccine to be made available, new promising direct-acting antiviral (DAA) drugs offer a better therapeutic scenario than in the past even for the poor responder genotypes 1 and 4.

## Transmission routes and epidemiology

Similarly to other parenterally-transmitted infections such as HIV and HBV, other various modalities of HCV transmission have been documented:

*- Intravenous drug use*. Since the most efficient transmission route of hepatitis C virus is percutaneous exposure, it is not surprising that intravenous needle sharing drug users show high infection rates, that may be as high as 90% when HIV co-infected drug addicts are considered [3].

*- Non-intravenous recreational drug exposure*. Increasing evidence is accumulating that HCV may also cross the nasal mucosa and infect subjects chronically using inhalatory recreational drugs, such as cocaine, by the sharing of inhalatory instrumentation, favored by the frequent bleeding of the nasal mucosa occurring in these individuals [4].

*- Accidental exposure*. The risk of HCV infection after accidental needle stick exposure has been reported to range between 0.2% to 10%, depending on various factors including hollow-bore needles, percutaneous exposure, high HCV viral load or HIV co-infection of the index case [5].

*- Healthcare procedures*. Exposure to unsafe healthcare practice, including hemodyalisis, has been reported to be one of the most important risk factors associated with HCV infection, even in western countries [6,7].

*- Mother to child vertical transmission*. Mother-to-child vertical transmission of HCV is reported to occur in 3-10% of cases, mostly in the late intrauterine period, at delivery or in the peri-natal period. Many factors have been reported to influence the transmission rate, including maternal high viral load, labour duration, newborn gender, premature membrane rupture and genotype [8]. The role of elective cesarean section to reduce mother-to-child transmission rates is debated and controversial [8,9] and the guidelines of the European Association for the Study of the Liver (EASL) does not recommend cesarean section to prevent HCV vertical transmission [10].

*- Sexual exposure*. The efficiency of the sexual transmission of HCV has been the subject of extensive debate and it is generally considered to be very low [11]. However, among male intravenous drug users, the rate of HCV infection was found to be one third higher in those who had sex with men (MSM) than in heterosexuals [12]. Recent evidence points to the increasing incidence of HCV infections in MSM, probably facilitated by rectal mucosa traumatisms, especially when HIV-infection co-exists. Paradoxically, it has been noted that the increased incidence of the sexual transmission of HCV has paralleled the increased availability of Highly Active Anti-retroviral Therapy (HAART), suggesting that a false-security feeling may have played a role [13].

However, for a large share of cases, estimated at around 30%, no definite exposure source may be identified. Studies of age-specific prevalence rates of anti-HCV in the population show that HCV infection is rare among children while it increases with age, suggesting a possible cohort effect in anti-HCV positive elderly individuals who acquired the infection several years ago, before the introduction of effective preventive measures such as screening of donors and use of disposable needles and syringes in medical practice. During the last 10 years, the rate of transfusion-associated hepatitis C has significantly dropped as a consequence of the introduction of increasing restrictions on donor eligibility and the implementation of effective anti-HCV or HCV-RNA screening. Consequently, most of the millions who are chronically infected with HCV are now in the fourth or fifth decade of life (“baby boomers”). As they move into their 60s and 70s, these individuals constitute a wave of asymptomatic HCV infection that may move toward clinical disease. Whatever the transmission route, it is estimated that approximately 170 million individuals, i.e. 3% of the world population, are chronically infected with HCV [14]. HCV is the primary cause of death of 350,000 individuals every year, also representing the primary reason for liver transplantation.

The prevalence varies markedly from *low* (< 2.5%) in North America, Europe, Australia and Far East, to *intermediate* (2.5% to 10%) in some Mediterranean countries, South America, Africa and Middle East to *high* (>10%) *prevalence* areas in Egypt, Burundi, Gabon, Cameroon, Rwanda, Guinea, Bolivia, Mongolia with an steady North-South increasing trend. It has been estimated that in Southern Europe 9 million persons are anti-HCV positive, 1,600,000 of whom in Italy, and that HCV accounts for approximately 20% of reported cases of acute hepatitis. Six genotypes, numbered 1-6, and a large number of subtypes have been described. Genotype 1 (subtype 1a and 1b) is by far the most prevalent genotype worldwide. A number of studies have reported that subtypes 1a and 1b predominates in America and Europe, and that subtype 1b is the predominant genotype in Asia. Both types 2 and 3 are found with significant prevalence in many countries in North and South America, Europe and Asia. Others studies have found type 4 to be predominant in Africa. However, HCV type 4 and 5 can also be found sporadically outside of Africa.

## Natural history and clinical impact

After HCV acute infection an average 50-85% of patients will not clear the virus, with higher rates in HIV-co-infected subjects, and will therefore remain chronically infected with *plateau* or fluctuating viremia detectable in the blood. The remaining 15-50% will gradually show a decrease and final disappearance of the virus from the blood, usually within 3 months from infection [15,16]. The complex mechanisms regulating virus clearance and persistence are still not completed understood, but probably imply both host and virus factors. The role of *ethnicity* has not been proved. On the contrary, *sexual transmission* of HCV and *HBV co-infection* might favor viral clearance, probably due to limited *inoculum* and viral interference, respectively [17]. From the virological perspective, the higher the genetic diversity of the infecting virus, the higher the probability that the immune response will not be able to control its replication, resulting in chronic infection, while a narrow quasispecies spectrum is more likely associated to viral clearance [18]. Of note, similar to hepatitis B infection but without genomic integration, it has been recently demonstrated that HCV may replicate in the liver in the absence of detectable viral level in the blood , a condition sometimes referred to as “occult C hepatitis”, with lower potential for progressive disease [19].

In the setting of persistent hepatitis C viremia, liver fibrosis is the consequence of chronic inflammation leading to the final distortion of hepatic architecture and impairment of liver microcirculation and cell functions. The main consequence of chronic HCV infection is the progression to cirrhosis, often clinically silent apart from non-specific symptoms such as fatigue, upper right quadrant pain or, sometimes, arthralgia and myalgia, until severe complications develop.

In most cases, abnormal ALT values are the only clinical aspecific findings of the disease, only representing a marker of hepatocellular dysfunction. In particular, a direct correlation between the degree of ALT elevation and stage of the HCV-related disease is often lacking as a significant cytolytic activity is not a surrogate marker of disease severity [20] as well as normal ALT does not always mean an healthy liver. Population-based studies indicate that up to 30%-40% of individuals with chronic HCV have persistently normal ALT values when serially tested. However, significant liver disease, with active inflammation and/or at least significant fibrosis, is biopsy-proven in about 20% of HCV carriers with normal ALT [21].

Chronic hepatitis C is the most common cause of cirrhosis and the most common indication for liver transplantation in Europe, North and South America, Australia and Japan. The risk of developing cirrhosis ranges from 5% to 25% over periods of 25-30 years [22].

Environmental and host factors can increase the risk and/or accelerate the natural course of HCV-related disease. Multiple studies have shown that *alcohol consumption*, in particular a daily intake greater than 40-50 g, is one of the most influential factor driving fibrosis progression in patients with HCV. *Age at time of infection* also plays a role: the estimated probability of progression is significantly higher in patients that were infected at an older age (> 40 years) [23]. Also, a recent and large analysis of published studies suggests that early acquisition of HCV in childhood is rarely associated to a severe future course of the disease [24]. Other factors that affect the progression of hepatic fibrosis include *male gender*, the *degree of inflammation* and *fibrosis* on the liver biopsy, co-morbidities such as *immunosoppression* or metabolic condition such as *non-alcholic steatohepatitis*, *obesity* and *insulin resistance *[20].

In addition, co-infection with HBV or HIV are significant risk factors for liver fibrosis. Approximately 4 to 5 million subjects with chronic hepatitis C are co-infected with HIV. Highest co-infection rates are observed among injection drug users (IDU): in the USA and in Europe, among HIV-infected IDU, HCV prevalence may be as high as 70-90 %. Paradoxically, the longer life-expectancy offered to HIV-infected patients by HAART permits slow-acting HCV-related liver injury to emerge as a significant cause of morbidity and mortality in HIV-HCV co-infected patients. Furthermore, the progression rate to cirrhosis and end-stage liver disease is accelerated in HIV co-infected patients: they have a twofold increased rate of cirrhosis compared to HCV mono-infected individuals [25], particularly when HIV associated immune-depression progresses.

The mechanisms underlying accelerated liver disease in HIV-HCV co-infected patients are not completely understood, possibly including direct HCV effects on hepatocytes and hepatic stellate cells as well as immunological alterations such as immune activation, apoptosis and impaired HCV specific T-cell response [26]. Furthermore, liver toxicity of anti-retroviral drugs and the burden of metabolic diseases may contribute to a faster progression of liver fibrosis in HIV-HCV co-infected patients.

Conversely, the role of HCV on the natural history of HIV infection continue to be debated and contrasting evidence exist [27,28].

HCV replication has been observed in extra-hepatic tissues, such as bone marrow, the central nervous system, endocrine glands, lymph nodes, spleen, monocytes, macrophages and skin cells. HCV is also often associated with profound alterations in the host immune system, resulting in immunological abnormalities and even autoimmune disease such as mixed cryoglobulemia (MC), rheumatoid factor (RF) production, B cell lymphoproliferative disorders that may progress to non–Hodgkin lymphoma, and others. Cryoglobulins are immunoglobulins that precipitate in the cold and are classified into three groups, based on Ig clonality. *Type I cryoglobulins* are usually associated with lymphoproliferative disorders, including myeloma and Waldenstrom macroglobulinemia, and usually consist of monoclonal IgM or IgG, rarely IgA. *Type II cryoglobulins* are composed of polyclonal IgG and monoclonal IgM, usually characterizing the condition known as *essential MC* that is often associated with HCV. *Type III MC* is also characterized by RF activity, although polyclonal IgG and polyclonal IgM exist. The incidence of HCV infection in MC ranges from 40% to 90%, with geographical variations [29]. The high incidence of disease among Mediterranean people and the association of certain human leukocyte antigen (HLA) supports that genetic factors play a role in the disease. The clinical picture is characterized by the skin manifestations ranging from purpura of lower limbs to chronic torpid skin ulcers, more frequent in the sovramalleolar regions. Skin reactions include Raynaud’s phenomenon, *livedo reticularis*, urticaria, and edema. Arthralgias more frequently involve the hands and the knees symmetrically. Renal injury may complicate MC in almost 30% of cases and involvement of the nervous system from 17% to 60%. Peripheral sensory-motor neuropathy can represent the first clinical sign of cryoglobulinemia.

## Cirrhosis and hepatocellular carcinoma (HCC)

An important clinical feature of HCV infection is the high rate of chronic and slowly progressive lifelong infection, which may lead to cirrhosis and end-stage liver disease in about 10-40% of people with chronic hepatitis C, depending on the presence of co-factors.

*Cirrhosis* is defined as the progressive development of regenerative nodules embedded in fibrous bands in response to chronic liver injury that leads to portal hypertension and end-stage liver disease [30]. Globally, 57% of cirrhosis is attributable to either HBV (30%) or HCV (27%), while alcohol consumption is another important cause, accounting for about 20% of the cases [2]. Alcoholic liver disease and hepatitis C prevail in western countries, whereas hepatitis B is the prevailing cause in most parts of Asia and sub-Saharan Africa [30]. Liver cirrhosis is a widespread chronic disease in Italy, where it was listed between the top-ten main causes of death in 2001. Survival of patients with cirrhosis is heavily influenced by the onset of complications (i.e., ascites, encephalopathy, jaundice, oesophageal varices bleeding), that occur at a yearly rate of about 5-7% patients.

Ascites occurs in at least 50% of the patients during their life, heralding a negative prognosis in the short-medium term. Elimination of the causal factor delays progression and could even reverse cirrhosis, even if data about reversibility are contrasting.

Liver cirrhosis is a major risk factor for the development of *hepatocellular carcinoma* (HCC). Indeed HCC is the most severe complication of chronic inflammatory liver diseases and it is the most frequent primary liver cancer. As an example, in Italy HCC accounts for as many as 79% of liver cancers [31]. The incidence of HCC increases progressively with advancing age in all populations, reaching a peak at 70 years, with a strong male preponderance [32]. The prevalence of HCC in Italy is 53/100000 and 22/100000 inhabitants in males and in females respectively (risk ratio 2:1). It is the fifth cause of death in men (third cause in the age group 50-69 years) and the seventh cause of death in women [31]. Recently, the mortality rate has shown a decrease of 34% in men and 41% in women in the period 2000-2009 in Italy [33], probably due to an overall improvement of management of HCC both in terms of early diagnosis and therapy. On a global scale, the most frequent causes of HCC are HBV (54%), HCV (31%) and alcoholic abuse (15%) [34]. In Africa and Asia an important role of co-factor in the HBV carcinogenesis is played by dietary exposure to aflatoxin B.

As 90% of HCC are associated with a known underlying risk factor it is possible to identify patients at high risk for developing HCC and enter them into surveillance programs aimed at early detection of neoplastic lesions in order to reduce disease-related mortality. Periodic ultrasound (US) scan is probably the most appropriate surveillance test, even if the outcome is highly dependent on the expertise of the examiner and on the physical typology of the patient [35]. Six-monthly surveillance appears to offer the best cost-benefit ratio [36].

**Figure 1:**
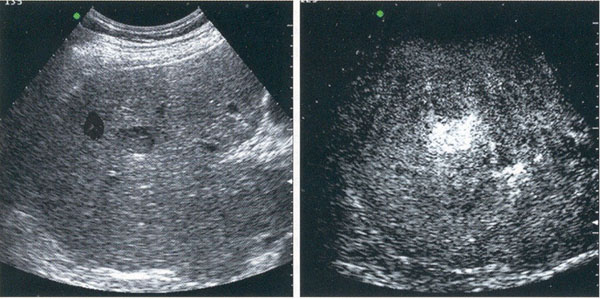
Left: Ultrasound (US) image of HCC. Right: contrast-US image of HCC with arterial enhancement (Courtesy Dr L. Biasi).

If histology remains the gold-standard, diagnosis may also be reached with high degree of specificity through non-invasive techniques (contrast TC or MRI), performing a 4-phase assessment of the lesion (pre-contrast, arterial, venous and delayed-contrast phase). Also contrast-US may efficiently lead to the diagnosis of HCC, provided that it is performed by highly skilled examiners (Figure. 1). HCC staging is based on CT or MRI images, but MRI is to be preferred when small lesions (Ø < 2 cm) are present [37]. In the absence of a universally accepted staging system, the current AASLD and EASL guidelines endorse the 5-stage Barcelona-Clinic Liver Cancer (BCLC) classification systems [38] because it links tumor stage with treatment strategy and includes prognostic variables related to tumor status, liver function and health performance status along with treatment-dependant variables.

## Public health impact of hepatitis C and its complications

The burden of HCV-related cirrhosis and HCC on the health systems of western countries is expected to increase in the coming years due to the diseases progression wave of HCV baby boomers, posing an increasing high burden on hepatologists and infectious diseases specialists.

In Europe, nine million people are affected by chronic hepatitis C and 86,000 people die each year because of HCV infection. In this continent, about 60-70% of HCC cases are caused by HCV and data from several european countries suggest that the mortality from liver cancer is rising [39].

In particular, Italy is the European country with the largest number of people chronically infected by hepatitis C virus [40], which is the leading cause of hepatic disease in this country. In Italy, chronic liver diseases impact on National Health System with about 160,000 hospital discharge diagnosis and account overall for 5% reimbursement due by the Regions to the Hospitals [41].

The Liver Match, an observational cohort study that prospectively enrolled liver transplantations performed at 20 Italian transplant Centers between 2007 and 2009, reported that hepatitis virus-related end stage liver disease (with and without HCC) accounts for 64.2% of indications to transplantation and HCV is the most frequent etiologic factor; in addition, 54% of the cases of HCV-related cirrhosis is associated with HCC [42].

## Screening strategies

As most HCV-infected individuals are unaware of their infection status, they are therefore not monitored nor offered expert advice or treatment, when needed. The knowledge of the basic drivers of HCV infection (risk factors, geographical origin, age groups, etc.) may provide the rationale for screening strategies aimed at detecting those individuals who might benefit from the new therapeutic advances. Most health authorities have recommended HCV screening in those individuals more likely to be infected [43]. When individuals at high-risk for HCV infection have been screened, high HCV prevalence have been indeed detected, as expected. However, the efficacy of this risk-based strategies has proved far from optimal and many patients, even those with recognized high risk of exposure, remain unscreened and undetected even in western countries. Furthermore, it has been estimated that as many as 30-45% of HCV-infected patients in western countries do not have significant risk factors nor elevated transaminase levels, so that they are missed by targeted screening. When blind screening to the general population attending large Emergency Departments in Germany have been carried out, a higher HCV seroprevalence than expected has been documented [44]. Recently, the cost-benefit to enlarge the criteria for HCV screening in order to detect a larger number of HCV-infected individuals has been addressed and found favorable in the medium term provided that referral, treatment and cure were made available. In this model simulation, the incremental cost, compared to risk-based guidelines, for every quality-adjusted life year (QALY) gained was 7,900 $ and 4,200 $ if once-time HCV screening was to be proposed to the 20-69 yrs old or to the *baby-boomers* (birth date from 1945 to 1965) US population, respectively [45].

On the basis of the growing epidemiological evidences, the US Department of Health and Human Services has recently issued the 2012 “Recommendations for the identification of chronic hepatitis C virus infection among persons born during 1945-1965” [46] recommending HCV screening testing for adults born in the period 1945-65 (one-time testing) in addition to testing adults of all ages reporting risk factors for HCV infection.

## Competing interests

The Authors declare that they have no conflict of interest related to the content of the specific article.

## Declarations

Publication of this supplement was partly supported by an unrestricted grant provided by Roche. The articles were independently prepared by the authors with no input from Roche. Roche were not involved in selecting the articles for the supplement.
